# Investigating the impact of Wnt pathway-related genes on biomarker and diagnostic model development for osteoporosis in postmenopausal females

**DOI:** 10.1038/s41598-024-52429-1

**Published:** 2024-02-04

**Authors:** Jinzhi Lai, Hainan Yang, Jingshan Huang, Lijiang He

**Affiliations:** 1 Department of Oncology, The Second Affiliated Hospital of Fujian Medical University, Quanzhou, 362000 Fujian China; 2 Department of Ultrasound, First Affiliated Hospital of Xiamen University, Xiamen, 361003 Fujian China; 3 Department of General Surgery, The Second Affiliated Hospital of Fujian Medical University, Quanzhou, 362000 Fujian China; 4 Department of Orthopaedic Surgery, The Second Affiliated Hospital of Fujian Medical University, Quanzhou, 362000 Fujian China

**Keywords:** Computational biology and bioinformatics, Genetics, Biomarkers, Endocrinology, Risk factors

## Abstract

The Wnt signaling pathway is essential for bone development and maintaining skeletal homeostasis, making it particularly relevant in osteoporosis patients. Our study aimed to identify distinct molecular clusters associated with the Wnt pathway and develop a diagnostic model for osteoporosis in postmenopausal Caucasian women. We downloaded three datasets (GSE56814, GSE56815 and GSE2208) related to osteoporosis from the GEO database. Our analysis identified a total of 371 differentially expressed genes (DEGs) between low and high bone mineral density (BMD) groups, with 12 genes associated with the Wnt signaling pathway, referred to as osteoporosis-associated Wnt pathway-related genes. Employing four independent machine learning models, we established a diagnostic model using the 12 osteoporosis-associated Wnt pathway-related genes in the training set. The XGB model showed the most promising discriminative potential. We further validate the predictive capability of our diagnostic model by applying it to three external datasets specifically related to osteoporosis. Subsequently, we constructed a diagnostic nomogram based on the five crucial genes identified from the XGB model. In addition, through the utilization of DGIdb, we identified a total of 30 molecular compounds or medications that exhibit potential as promising therapeutic targets for osteoporosis. In summary, our comprehensive analysis provides valuable insights into the relationship between the osteoporosis and Wnt signaling pathway.

## Introduction

Osteoporosis, a prevalent systemic bone disease, is characterized by a decrease in bone mineral density (BMD) and deterioration of bone structure, thus increasing the susceptibility to bone fragility and fractures^1^. Although it often remains asymptomatic in the early and middle stages, acute osteoporotic fractures can lead to long-term disability and a higher risk of fractures^2^. With an aging population, social costs related to osteoporosis are expected to increase. Almost 40% of women experience osteoporosis and fragility fractures during their lifetime^3^. Consequently, the early detection and treatment of osteoporosis significantly enhance patients’ survival and quality of life ^4^. Therefore, identifying diagnostic and therapeutic biomarkers with high sensitivity and specificity is crucial for improving management practices for osteoporosis.

The Wnt signaling pathway plays a pivotal role in the differentiation of osteoblasts and the inhibition of osteoclasts, which are vital for skeletal development and adult skeletal homeostasis^5^. Several studies reported that mechanical loading triggers Wnt signaling in cells of the osteoblastic lineage, indicating that Wnt activity might be responsible for connecting mechanical forces to an anabolic response in the skeletal system^6^. The Wnt pathway is crucial for bone formation, as genetic alterations in its components have been associated with various skeletal disorders^7^. These mechanisms could potentially contribute to the pathogenesis of disuse-related osteoporosis. For instance, inhibiting sclerostin, a Wnt antagonist secreted by osteocytes, can increase osteoblast activity and bone formation, ultimately enhancing bone strength^8^. Despite substantial evidence linking bone disorders with the Wnt pathway, the specific genes and molecular interactions within these networks remain unclear. Moreover, no studies have reported the correlation between osteoporosis and Wnt pathway-related genes. Recently, there has been increasing recognition of several genes associated with osteoporosis as potential biomarkers for diagnosis^9–11^. Besides, researchers have also explored the use of gene sets or pathways as biomarkers for osteoporosis^12,13^. These studies provided a more comprehensive understanding of the molecular mechanisms underlying osteoporosis. Therefore, it is important to investigate molecular clusters associated with the Wnt signaling pathway and construct a diagnostic model for osteoporosis focused on Wnt pathway-related genes.

BMD testing is widely used to diagnose and evaluate fracture risk for osteoporosis^14^. In our study, we analyzed the expression profiles of Wnt pathway-related genes in patients with low and high BMD using publicly available Gene Expression Omnibus (GEO) datasets. We segregated osteoporosis patients into two distinct clusters using a consensus clustering method. To construct a diagnostic model, we compared four machine learning models based on 12 osteoporosis-associated Wnt pathway-related genes. Among these models, the extreme gradient boosting (XGB) model showed the most promising discriminative potential. We then constructed a diagnostic nomogram based on the five hub genes identified from the XGB model. Moreover, we utilized the Drug Gene Interaction Database (DGIdb) to identify drugs and molecular compounds that interact with five hub genes. Through our investigation of the association between Wnt pathway-related genes and osteoporosis, we have successfully developed a diagnostic model that can accurately assess the risk of osteoporosis in patients. These findings enhance our understanding of the underlying pathogenesis of osteoporosis and offer potential therapeutic targets for further investigation.

## Materials and methods

### Data acquisition and BMD measurements

In this study, we utilized three microarray datasets (GSE56814, GSE56815, GSE2208) from the GEO website database (www.ncbi.nlm.nih.gov/geo) as training sets to investigate the relevance of osteoporosis to gene expression profiles^15–17^. These datasets comprised 26 high BMD and 16 low BMD patients, 20 high BMD and 20 low BMD patients, and 5 high BMD and 5 low BMD patients, respectively. The hip BMD (g/cm2) of each subject was measured using a Hologic dual-energy X-ray absorptiometer (DXA) scanner and transformed into a Z score using a healthy, ethnic-, gender-, and age-matched reference population. The high BMD group was defined as Z_BMD_ > + 0.84, while the low BMD group was defined as Z_BMD_ < − 0.52. Individuals with diseases that might affect bone metabolism were excluded using strict exclusion criteria, and circulating monocytes were used as the sample tissue.

To standardize the data and remove batch effects in these datasets, we employed the 'Surrogate Variable Analysis (sva)' R package (https://bioconductor.org/packages/release/bioc/html/sva.html). We then utilized the 'Linear Models for Microarray Data (limma)' R package to screen for differentially expressed genes (DEGs) in the training set (https://bioconductor.org/packages/release/bioc/html/limma.html), using the inclusion criteria of |log2-fold change (FC)| > 0.1 and adjusted p-value < 0.05. Additionally, we employed three independent datasets (GSE7158, GSE7429, and GSE13850) as validation sets, which were previously used in several monocyte microarray studies for osteoporosis^18^. Furthermore, we downloaded 329 Wnt pathway-related genes from the Reactome pathway database (https://reactome.org/) to investigate the impact of Wnt pathway-related genes on osteoporosis.

### Gene set variation analysis (GSVA) and pathway activity analyses

To evaluate the variation in pathway activity across different groups, we conducted functional annotation and pathway enrichment analyses. Gene Set Variation Analysis (GSVA) was performed in an unsupervised manner using the 'GSVA' package^19^. For this analysis, we obtained the gene set 'c2.cp.kegg.v7.5.1.symbols.gmt' from the Molecular Signatures Database (MSigDB) and selected it as the background gene set^20^. To identify biological pathways and processes, we employed Gene Set Enrichment Analysis (GSEA). The gene set 'c5.go.v7.5.1.symbols' was retrieved from the MSigDB and chosen as the background gene set for this analysis^21^. A p-value of less than 0.05 and a q-value of less than 0.05 were considered significant.

### Immune cell infiltration analyses

In order to assess the composition of tumor-infiltrating immune cells in our samples, we employed CIBERSORT, a computational tool that utilizes gene expression data to estimate the relative abundances of different immune cell types within a mixed cell population^22^. This analysis was conducted using the 'CIBERSORT' R package. A p-value threshold of ≤ 0.05 was applied to determine the statistical significance of the results and to select samples for further analysis.

### Unsupervised consensus clustering analysis

To investigate the clustering of osteoporosis patients based on the expression of DEGs related to the Wnt pathway, an unsupervised consensus clustering approach was employed using the 'Consensus Cluster Plus' R package. The analysis was performed with 1000 iterations, where each iteration resampled 80% of the data. To determine the optimal number of categories, we utilized cumulative distribution function (CDF) curves and consensus matrix (CM) plots.

### Development of a diagnostic model employing four machine learning methods.

We used four machine-learning predictive models to investigate the association between gene expression and osteoporosis. These models included the support vector machine (SVM), random forest (RF), generalized linear model (GLM), and XGB model. The SVM algorithm seeks to identify a hyperplane that maximizes the margin between positive and negative instances, enabling effective differentiation^23^. The RF approach utilizes multiple decision trees, each providing independent predictions to classify or regress outcomes^24^. GLM extends the multiple linear regression model, allowing for flexible assessment of the relationship between normally distributed dependent characteristics and continuous or categorical independent characteristics^25^. XGB, on the other hand, consists of a collection of gradient-boosted trees that meticulously analyze complexity and classification errors^26^. To train and evaluate these models, we divided our dataset of 92 samples into a training set (70%, N = 67) and a validation set (30%, N = 25). The response variable was the categorization of low and high BMD, while the explanatory variables consisted of differentially expressed genes (DEGs) associated with the Wnt pathway. To optimize the performance of the models, we utilized the 'caret' package to tune their parameters through grid search, and evaluated their performance using fivefold cross-validation. To gain insights into the models and interpret their results, we employed the 'DALEX' package. This package allowed us to explain the four machine learning models used, analyze the distribution of residuals, and visualize the importance of features among the models.

### Prediction of the potential drugs

We utilized the Drug Gene Interaction Database (DGIdb, https://dgidb.genome.wustl.edu/), a comprehensive online resource that provides valuable information on the associations between genes and their known or potential drug interactions^27^. By leveraging this database, we could identify potential medications and chemical substances that interact with hub genes, thereby gaining insights into the molecular mechanisms underlying the pathogenesis of the disease under investigation. The cytoscape (version 3.7.2) was used to visualize and analyze the complex network of interactions between drugs the drug-gene interaction network^28^.

### Statistical analysis

We conducted all statistical analyses using R software version 4.1.3. To compare two groups with normally distributed data, we applied the Student's t-test. The chi-square test was employed to compare categorical and pairwise features across different groups. We utilized the Mann–Whitney *U* test to assess the presence of statistically significant differences between two groups, while the Kruskal–Wallis test was employed to evaluate statistically significant differences among multiple independent groups. To assess correlations between normally distributed variables, we employed Pearson's correlation test. For non-normally distributed variables, we utilized Spearman's correlation test. All statistical tests were two-sided, and we defined statistical significance as a p-value less than 0.05 unless otherwise specified.

## Results

### Identifying Wnt pathway-related genes associated with osteoporosis in postmenopausal females

**Figure 1:**
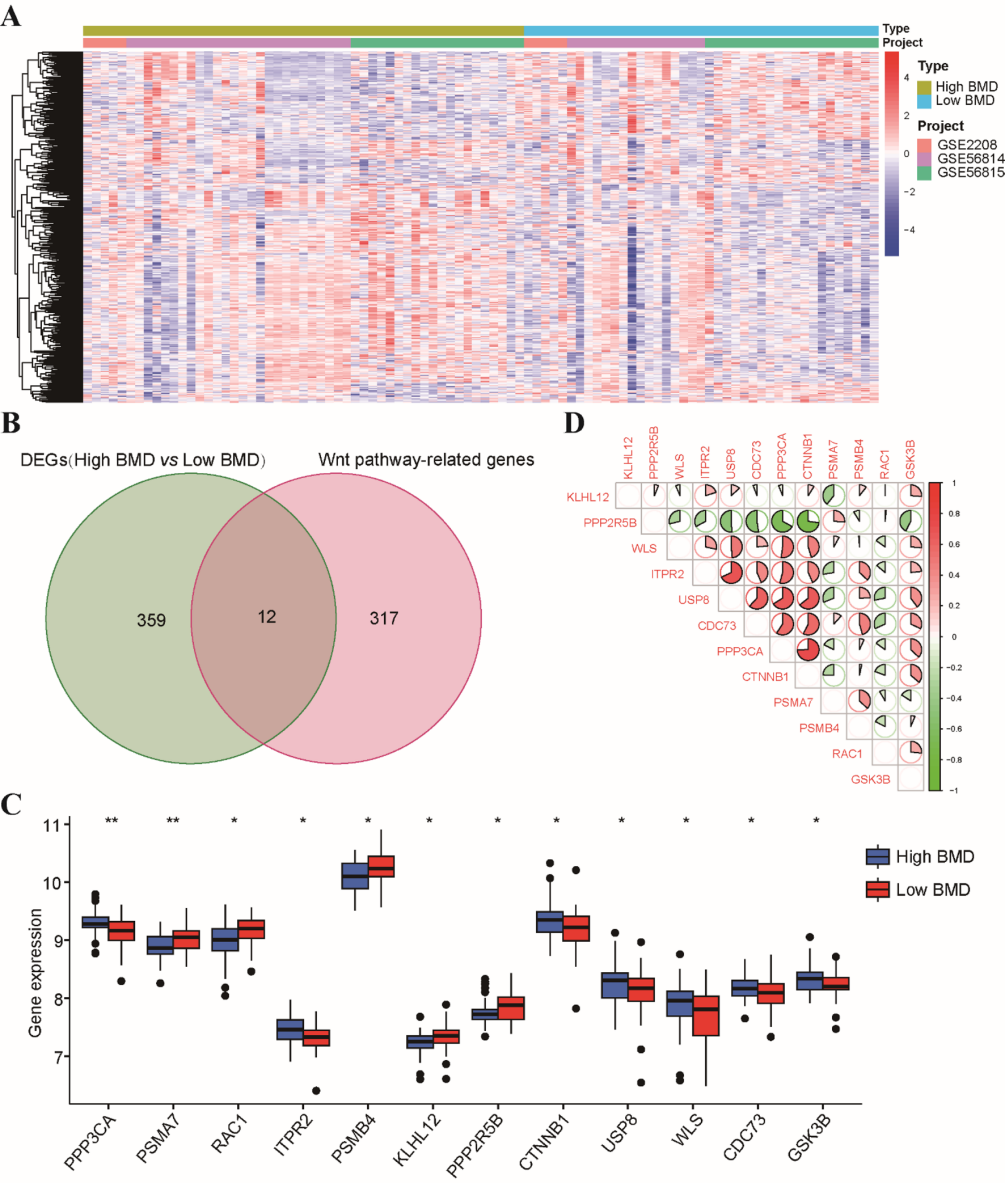
Identification of osteoporosis-associated genes associated with Wnt pathway. (**A**) The heatmap of the expression of 371 DEGs, where red indicates high expression and blue indicates low expression. (**B**) Venn diagram of intersections between 371 osteoporosis-associated gene and 329 related to Wnt pathway-related genes. (**C**) The expression levels of 12 osteoporosis-associated Wnt pathway-related genes were shown in the histogram. (**D**) The correlation analysis of 12 osteoporosis-associated Wnt pathway-related genes. *p < 0.05, **p < 0.01.

In this study, we utilized three datasets, namely GSE56814, GSE56815, and GSE2208 as the training set. The patients included in these datasets were postmenopausal unrelated Caucasian females, and detailed characteristics of the datasets can be found in Table S1. By implementing batch correction techniques, we observed a noticeable trend in the distribution of expression profiles across all samples (Supplementary Fig. S1A). We compared the low BMD group with the high BMD group to identify DEGs associated with osteoporosis, considering the inclusion criteria (|log2-fold change (FC)| > 0.1 and adjusted p value < 0.05). As a result, we identified a total of 371 DEGs between low and high BMD groups, including 138 up-regulated genes and 233 down-regulated genes (Fig. 1A). Among these DEGs, we found that 12 genes overlapped with the set of 329 Wnt pathway-related genes (Fig. 1B). Five of these 12 osteoporosis-associated Wnt pathway-related genes were up-regulated, while the remaining seven were down-regulated in the low BMD group (Fig. 1C). To comprehensively examine the genomic positions, annotations, and interrelationships of these 12 genes, we initiated a circos plot to illustrate their potential associations within the chromosomal framework (Supplementary Fig. S1B). Furthermore, we conducted correlation analysis to investigate the interactions among these osteoporosis-associated Wnt pathway-related genes. The application of a correlation cutoff at 0.3 ensured the focus on robust correlations while filtering out weaker associations (Fig. 1D and Supplementary Fig. S1C).

### Molecular pathways and tumor immune infiltration analyses between high and low BMD groups

**Figure 2:**
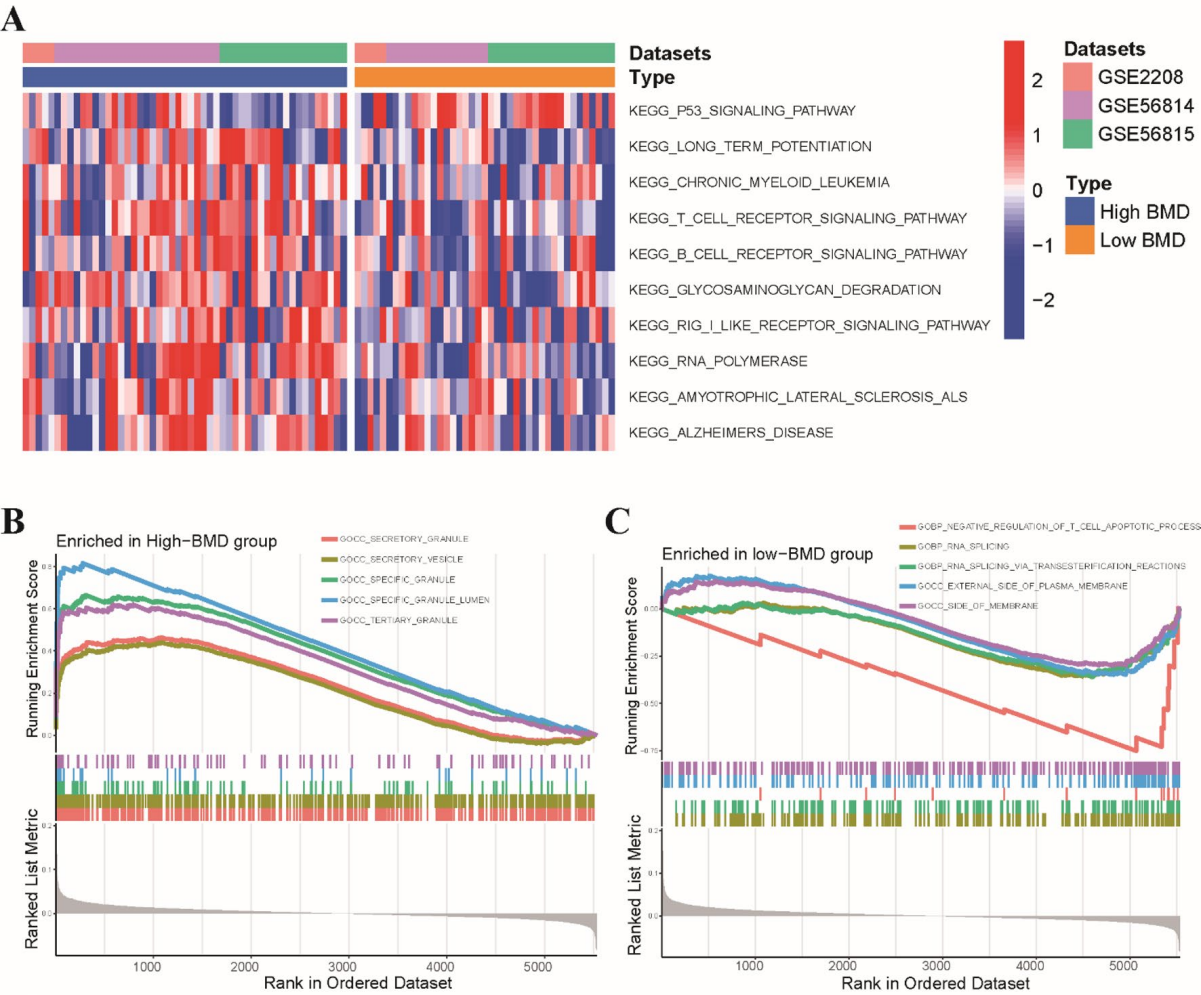
Functional annotations and tumor immune infiltration analyses of osteoporosis. (**A**) Heatmap illustrated the enrichment scores of top 10 differentially enriched KEGG pathways evaluated by GSVA analysis between high and low BMD groups. (**B**) GSEA showed that samples of the high BMD group were enriched in specific granule, secretory vesicle and tertiary granule. (**C**) Samples in the low BMD group were enriched in external side of plasma membrane and RNA splicing.

To provide a more comprehensive understanding of the molecular pathways and mechanisms underlying osteoporosis, we applied GSVA to identify signaling pathways distinguishing these two groups. Our results revealed that the low BMD group was mainly correlated with the p53 signaling pathway, while the high BMD group was predominantly associated with the glycosaminoglycan degradation pathway, long-term potentiation pathway, and B cell receptor signaling pathway (Fig. 2A). Subsequently, we used GSEA to further explore the pathway enrichment between high and low BMD groups. Our findings showed that patients in the high BMD group were mainly enriched in cellular components such as specific granule, secretory vesicle, and tertiary granule (Fig. 2B), whereas patients of the low BMD group were predominantly enriched in external side of plasma membrane and RNA splicing (Fig. 2C). These results suggested that the high and low BMD groups may exhibit distinct molecular pathways and cellular processes, which may contribute to the pathogenesis of osteoporosis.

**Figure 3:**
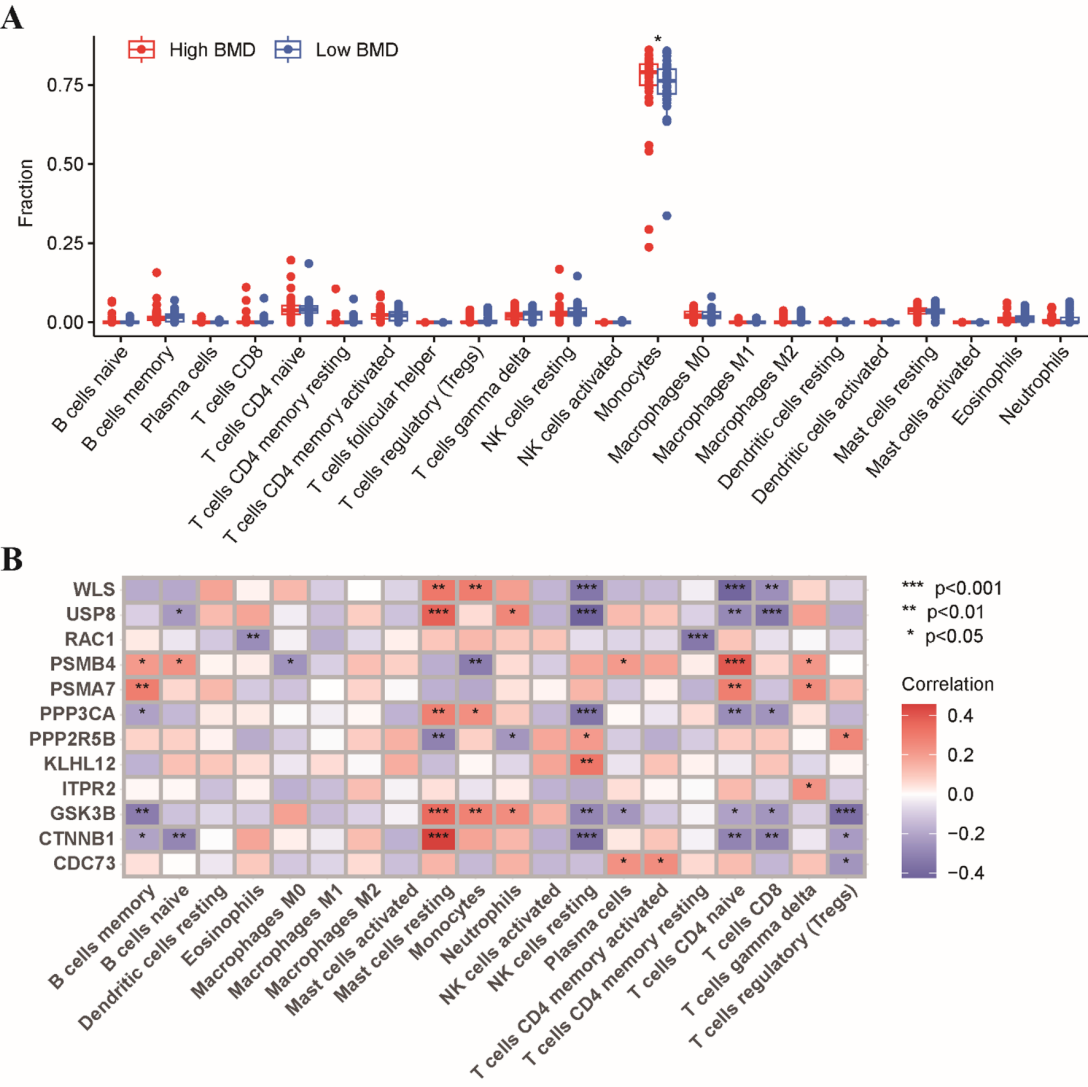
Relationship between osteoporosis-associated Wnt pathway-related genes and immune cells. (**A**) Comparisons of the proportions of 22 immune-infiltrating cells between these two groups. (**B**) The correlation analysis between 12 osteoporosis-associated Wnt pathway-related genes and immune cells. Red represents positive correlation; blue represents negative correlation. *p < 0.05, **p < 0.01, ***p < 0.001.

Next, we utilized the CIBERSORT algorithm to quantify the abundances of 22 immune-infiltrating cells between the two groups. Our results revealed that the abundance of monocytes was high in both two groups, with a significantly higher enrichment in the low BMD group (Fig. 3A). Then, we further analyzed the association between 12 Wnt pathway-related genes and immune cells. Of these genes, GSK3B, WLS, and PPP3CA had a significantly positive correlation with monocytes, while exhibiting a negative correlation with resting CD4 T cells, CD8 T cells and resting NK cells (Fig. 3B). These findings suggested that the Wnt pathway-related genes may be involved in modulating immune cell infiltration in the bone microenvironment.

### Identification of Wnt pathway-related molecular clusters

**Figure 4:**
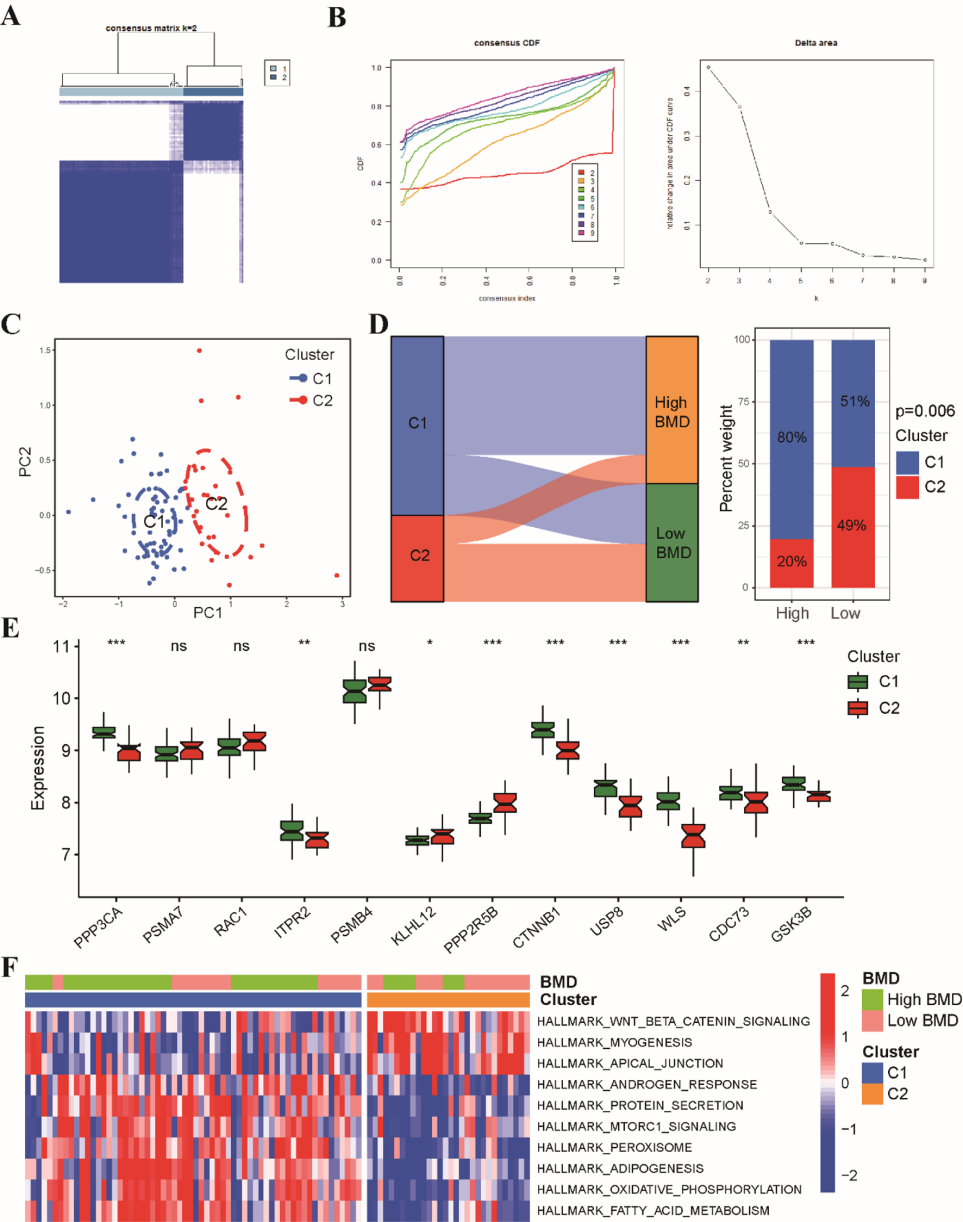
Identification of Wnt pathway-related clusters in osteoporosis. (**A**) The consensus matrix heat map identified three distinct clusters. (**B**) Representative CDF curves and CDF delta area curves confirmed the separation of clusters. (**C**) Principal component analysis (PC1 and PC2) further demonstrated the presence of distinct clusters. (**D**) The ggalluvial diagram and stacked histogram visualized the relationships between bone mineral density and cluster. (**E**) A boxplot displayed the expression patterns of 12 osteoporosis-associated Wnt pathway-related genes. (**F**) GSVA enrichment analysis revealed significant differences in signaling pathways between clusters 1 and 2. *p < 0.05, **p < 0.01, ***p < 0.001.

In order to explore the expression patterns of Wnt signaling pathway in osteoporosis, we utilized a consensus clustering algorithm to categorize patients based on the expression profiles of 12 Wnt pathway-related genes. We observed that the cluster numbers remained stable when the k value was set to two (k = 2), and the CDF curves exhibited minimal fluctuations within a consensus index range of 0.2 to 0.9 (Fig. 4A). We calculated the area under the CDF curve to determine the difference between two CDF curves (k and k − 1) for k values ranging from 2 to 9 (Fig. 4B). Additionally, at k = 2, the consistency score of all clusters exceeded 0.9 (Supplementary Fig. S2). Based on the consensus matrix heatmap, we classified the 92 patients into two clusters: cluster 1 (n = 62) and cluster 2 (n = 30). Subsequently, we subjected the patients to principal component analysis (PCA), which revealed a significant distinction between the two clusters (Fig. 4C). Further analysis demonstrated that most of patients in cluster 2 belonged to low BMD group, while the majority of patients in cluster 1 belonged to high BMD group (Fig. 4D). We examined the expression differences of 12 osteoporosis-associated Wnt pathway-related genes between clusters 1 and 2, and observed distinct expression patterns. Cluster 1 exhibited high expression of PPP3CA, ITPR2, USP8, WLS, GSK3B, and CTNNB1, whereas cluster 2 displayed up-regulation of KLHL12 and PPP2R5B (Fig. 4E). Pathway enrichment analysis, using hallmark gene sets, demonstrated that cluster 2 was mainly related to the Wnt/β-catenin signaling pathway, myogenesis, and apical junction pathways, whereas cluster 1 was principally associated with oxidative phosphorylation, fatty acid metabolism, and the protein secretion pathway (Fig. 4F).

### Machine learning model development for osteoporosis prediction

**Figure 5:**
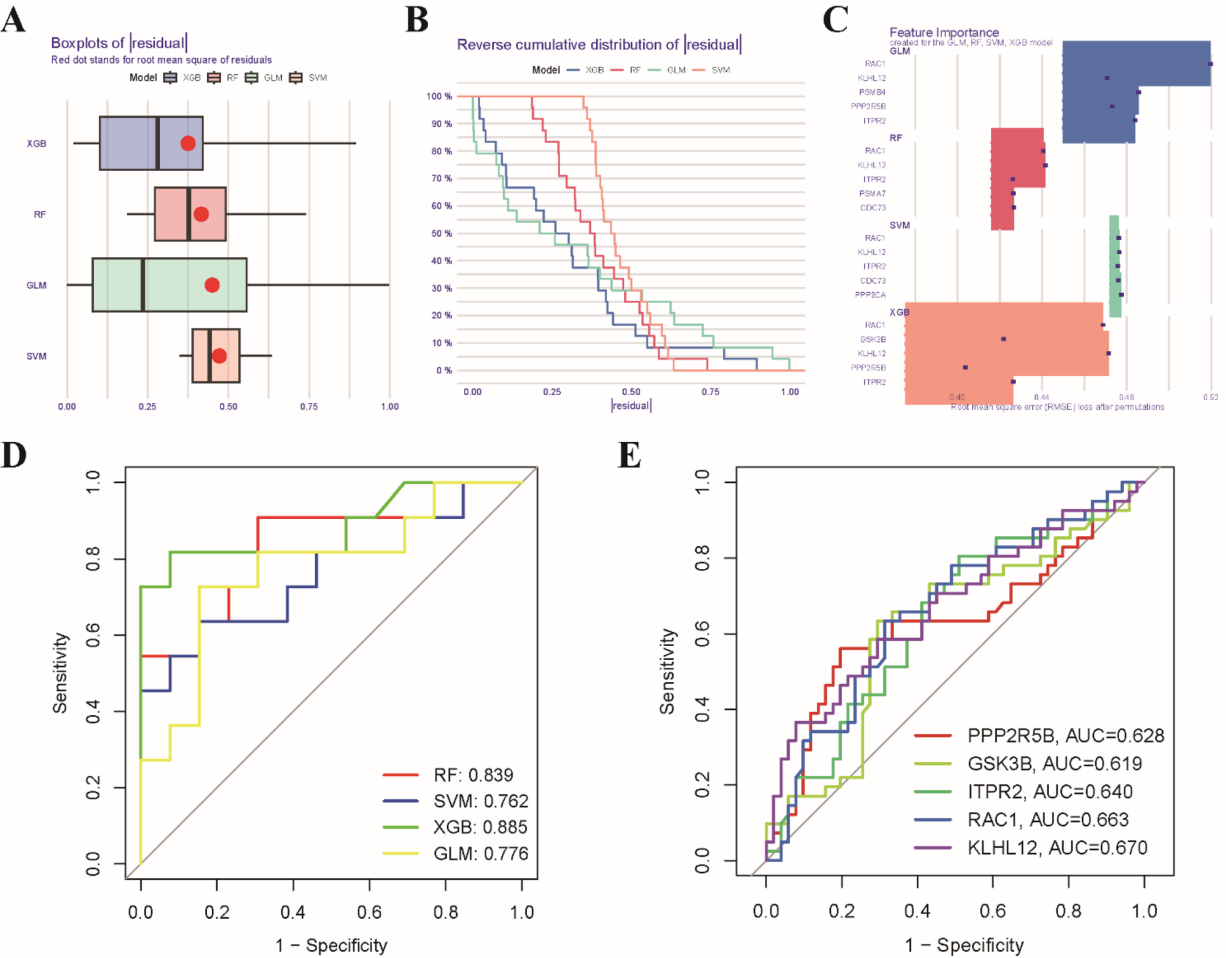
Construction and validation of a machine learning model for classification of osteoporosis. (**A**) Boxplots showing the residuals of each machine learning model. The red dot represents the root mean square of the residuals (RMSE). (**B**) A cumulative residual distribution plot of four machine learning models. (**C**) Identification of important features in SVM, RF, GLM, and XGB machine models. (**D**) ROC analysis of four machine learning models based on fivefold cross-validation in the testing cohort. (**E**) ROC analysis of the performance of 5 single genes using machine learning algorithms.

In order to identify critical markers with significant diagnostic value, we employed four distinct machine learning models to establish a diagnostic signature based on the expression profiles of 12 osteoporosis-associated Wnt pathway-related genes in the training cohort. The evaluation of residual distributions, using reverse cumulative distribution and boxplots, revealed that the XGB and RF machine learning models exhibited relatively lower residuals compared to the other two models (Fig. 5A,B). We determined the top 5 important variables for each model based on the root-mean-square error (RMSE) (Fig. 5C). Subsequently, the discriminative performance of the four machine learning algorithms was evaluated in the testing set through receiver operating characteristic (ROC) curve analysis, employing fivefold cross-validation. The XGB machine learning model demonstrated the highest area under the ROC curve (Fig. 5D). Hence, considering these results, the XGB model was determined to be the most effective in distinguishing patients. Finally, we selected the top five predictor genes (RAC1, KLHL12, GSK3B, ITPR2, and PPP2R5B) from the XGB model for further analysis. The ROC results for these 5 genes, using the machine learning algorithms, are presented in Fig. 5E.

### Nomogram construction and validation of diagnostic model

**Figure 6:**
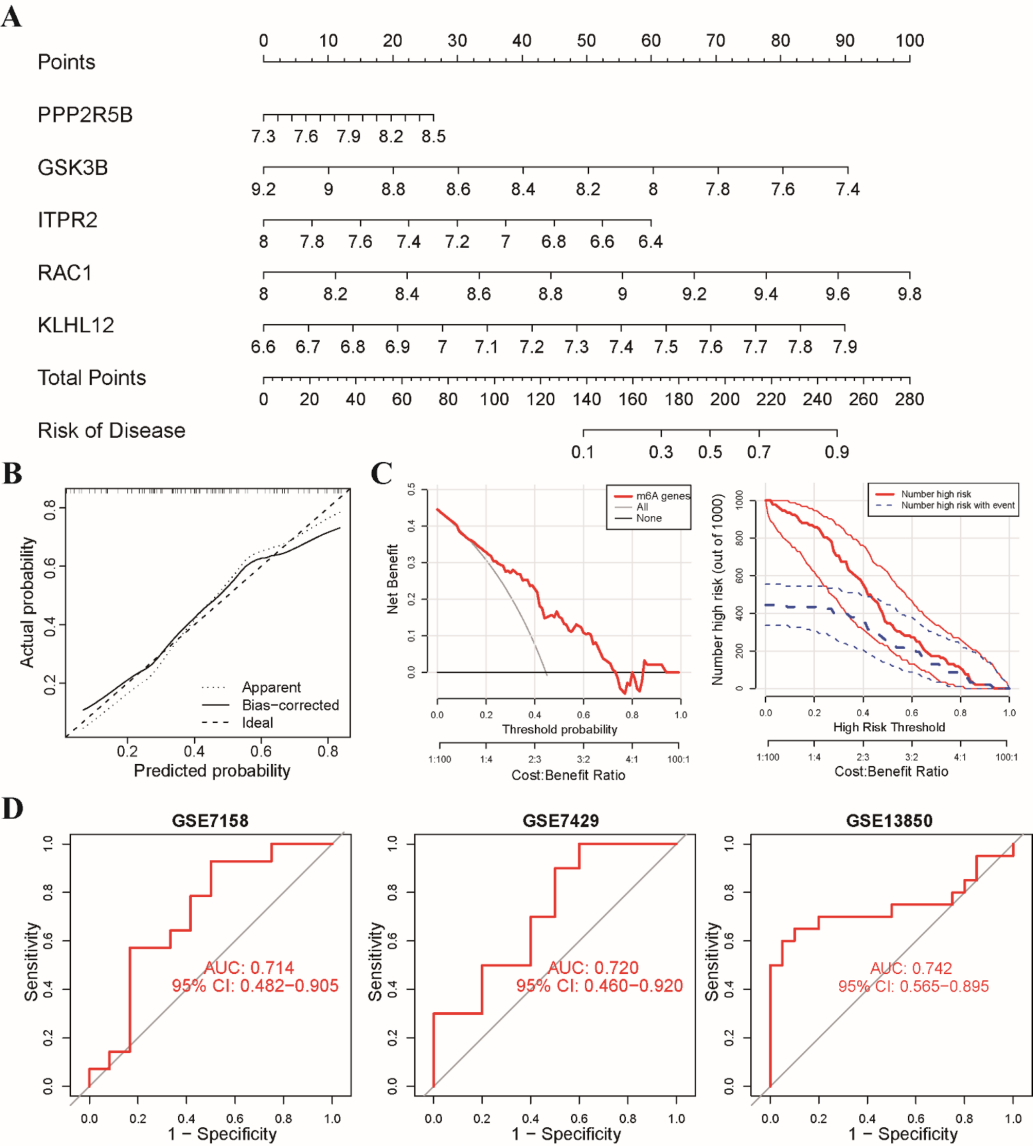
Development of the nomogram model for diagnosis of osteoporosis. (**A**) Construction of a nomogram model for osteoporosis diagnosis using a 5-gene-based XGB model. (**B**) A calibration curve showing the diagnostic accuracy of the nomogram model. (**C**) A decision curve analysis (DCA) and clinical impact curve (CIC) curve plot used to evaluate the predictive efficiency of the nomogram model. (**D**) ROC analysis of the 5-gene-based XGB model based on fivefold cross-validation in GSE7158, GSE7429, and GSE13850.

To assess the predictive efficiency of the XGB model further, we constructed a nomogram, which served as a valuable tool for facilitating the diagnosis of osteoporosis. The five most important genes from the XGB model, namely RAC1, KLHL12, GSK3B, ITPR2, and PPP2R5B, were selected as diagnostic markers to construct the nomogram (Fig. 6A). To evaluate the predictive efficiency of the nomogram model, we implemented calibration curve analysis, decision curve analysis (DCA), and clinical impact curve (CIC). The calibration curve demonstrated that the nomogram model accurately predicted the positive rate of osteoporosis with a high level of precision (Fig. 6B). DCA and CIC further confirmed the high accuracy of the model, providing strong evidence to support clinical decision-making (Fig. 6C). Additionally, we sought to validate the predictive capability of our diagnostic model using three external datasets (GSE7158, GSE7429, and GSE13850). The ROC curves depicted an impressive performance of the 5-gene diagnostic model, with an area under the curve (AUC) value of up to 0.7 across all three datasets (Fig. 6D). These results suggest that our diagnostic model exhibits equal efficacy in distinguishing osteoporosis from normal individuals, highlighting its potential clinical utility.

### Potential drug screening of hub diagnostic markers and interactions for osteoporosis

**Figure 7:**
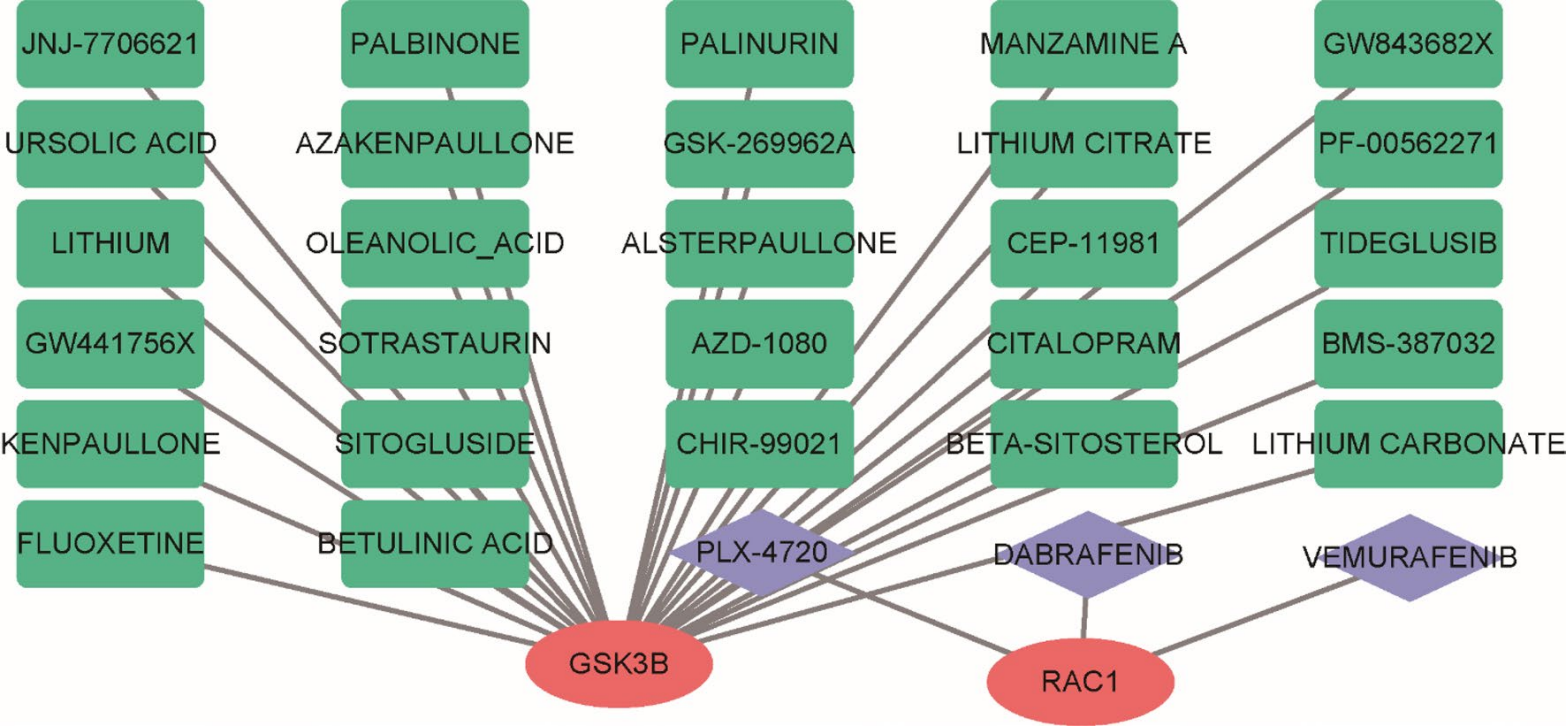
Prediction of targeted drugs for five diagnostic markers. Drug–gene interaction network based on the DGIdb database.

To identify potential therapeutic agents for osteoporosis, we utilized the DGIdb database to investigate drugs and molecular compounds that interact with five hub genes. The drug-gene interaction network revealed 30 molecular compounds or drugs that could be potential candidates. Among these, 27 molecular compounds or medications, including sotrastaurin, palbinone, β-Sitosterol, and fluoxetine, have been previously associated with GSK3B. Additionally, dabrafenib and vemurafenib were found to modulate the expression of the RAC1 gene (Fig. 7). These findings suggest that these drugs and molecular compounds may have the potential to target and modulate the activity of these hub genes, thereby offering potential therapeutic options for the treatment of osteoporosis.

## Discussion

In recent years, there has been significant advancement in our comprehension of the cellular processes and signals that govern bone remodeling. However, the specific targets and therapeutic mechanisms involved in treating osteoporosis remain incompletely understood and necessitate further investigation. A crucial element of these advancements is the pivotal role played by the Wnt signaling pathway in skeletal biology, which holds immense importance in both skeletal development and adult skeletal homeostasis^29^. Genetic modifications affecting various components of the Wnt signaling pathway in humans and animal models have served as the foundation for the development of innovative therapeutic strategies^30^. Consequently, early diagnosis and intervention at the molecular level in osteoporosis patients assume paramount clinical significance^31^. Low BMD, particularly in the hip and spine, is recognized as a primary contributing factor to heightened fracture risk. In our study, we conducted a gene difference analysis between groups with low and high BMD, investigating their correlation with the Wnt pathway. Employing cluster analysis, our results revealed that osteoporosis could be categorized into two distinct clusters. Through machine learning, we identified five potentially significant hub genes associated with the Wnt pathway. Furthermore, our investigation identified drugs and molecular compounds that interact with hub genes related to the Wnt signaling pathway in the context of osteoporosis. Overall, our study offers novel insights into the potential pathogenesis of osteoporosis and the therapeutic targeting of genes, with a particular emphasis on the relevance of the Wnt pathway.

The clinical diagnosis of osteoporosis has significantly improved due to the integration of bone biomarker measurements, such as BMD, and the application of machine learning modeling. Machine learning modeling has emerged as a valuable tool for analyzing complex data relationships and supporting clinical decision-making^32,33^. In contrast to traditional univariate analyses, machine learning approaches offer a more comprehensive understanding of the complex relationships between variables, resulting in improved accuracy and reliability. For instance, Xue et al. conducted a study where they developed a diagnostic model by incorporating six specific genes associated with osteoporosis. Their model achieved an impressive AUC value of 0.7265, indicating its high predictive power^34^. Similarly, Hwang utilized five different machine learning algorithms to develop models for osteoporosis screening. These models achieved AUC values ranging from 0.821 to 0.843 in men and 0.767 to 0.811 in women, further highlighting the effectiveness of machine learning in predicting osteoporosis^35^. In our study, we employed four machine learning classifiers (RF, SVM, GLM, and XGB) to analyze the expression profiles of Wnt pathway-related genes. Our models achieved an impressive AUC value of 0.853, a sensitivity of 82% and an accuracy of 71% in predicting osteoporosis, highlighting their potential as valuable tools for clinical decision-making. The performance of our model, including its sensitivity and specificity, was comparable to previous studies in the field^36^. It is worth noting that our study is the first to incorporate the Wnt signaling pathway in the prediction of osteoporosis. By integrating this pathway into our prediction models, healthcare professionals can potentially identify individuals at risk of osteoporosis at an earlier stage, enabling timely interventions and preventive measures.

Our findings revealed significant variations in the signaling pathways associated with these two groups. These observed differences may indicate discrepancies in bone remodeling and turnover rates between individuals with low and high BMD. These observed differences may reflect variations in bone remodeling and turnover rates between the two groups. The p53 signaling pathway, a well-known regulator of apoptosis and cell cycle control, plays a crucial role in maintaining bone formation and resorption^37^. On the other hand, the glycosaminoglycan degradation signaling pathway and B cell receptor signaling pathway are involved in promoting osteoblast differentiation and angiogenesis, both of which are essential for healthy bone growth and differentiation^38–40^. It is plausible to suggest that the disparities observed in these pathways could reflect variations in bone turnover and remodeling rates between individuals with low and high BMD. It is important to consider that several factors, such as ethnicity, age, lifestyle choices, and genetic predisposition, can influence these differences in bone remodeling and turnover.

Subsequently, we conducted an unsupervised cluster analysis to explore the regulation patterns of the Wnt pathway among individuals with osteoporosis. By analyzing the expression profiles of 12 osteoporosis-associated genes related to the Wnt pathway, we identified two distinct clusters within the osteoporosis patient population. Cluster 2 was predominantly composed of individuals with low bone mineral density (BMD) osteoporosis, while cluster 1 consisted mainly of individuals with high BMD osteoporosis. Our analysis revealed that cluster 2 was primarily associated with androgen response, oxidative phosphorylation, and fatty acid metabolism. The fatty acid metabolism and androgen response pathways are known to play crucial roles in regulating bone remodeling and maintaining the delicate balance between bone formation and resorption^41^. Therefore, the enrichment of these pathways in cluster 2 may contribute to the impaired bone remodeling observed in individuals with high BMD osteoporosis. These findings represent a significant advancement in our understanding of the molecular mechanisms underlying the development of osteoporosis. However, further investigations are warranted to elucidate the precise mechanisms through which these pathways influence BMD and overall bone health.

Our findings demonstrated that the XGB classifier exhibited superior predictive efficacy in training set, indicating its potential as a diagnostic tool for osteoporosis. Furthermore, we validated the performance of this model in two independent datasets and confirmed its high accuracy and specificity in predicting osteoporosis. Notably, we developed a nomogram based on the expression levels of five hub genes (RAC1, KLHL12, GSK3B, ITPR2, and PPP2R5B), which demonstrated significant predictive value and highlighted the clinical applicability of our approach. Among these hub genes, RAC1 has been implicated in enhancing osteoclast function in the context of Nf1 haploinsufficiency, suggesting its potential as a promising therapeutic target for osteoporosis^42^. Similarly, GSK3B has been identified as a protective factor against chondrocyte degradation through extracellular mechanisms. The AKT-GSK3β signaling pathway regulates the cleavage of optic atrophy 1, which is associated with mitochondrial dysfunction and may contribute to osteoblast apoptosis^43^. Overall, by targeting specific genes and pathways, we can potentially tailor treatments to individual patients, maximizing their effectiveness and improving patient outcomes.

The significance of the Wnt pathway in the development of osteoporosis has garnered considerable interest. Modulating this pathway shows potential as a therapeutic approach for individuals suffering from osteoporosis^5^. To identify potential therapeutic agents for osteoporosis, we employed the DGIdb database to explore molecular compounds and drugs that could potentially modulate the expression of hub genes implicated in osteoporosis. Notably, several molecular compounds and medications, including sotrastaurin, palbinone, β-Sitosterol, and fluoxetine, have been associated with GSK3B. Furthermore, dabrafenib and vemurafenib have demonstrated modulatory effects on RAC1 gene expression, suggesting their potential therapeutic relevance for osteoporosis. These findings underscore the importance of targeting these genes and their associated pathways in the development of innovative therapeutic approaches for individuals with osteoporosis. However, further extensive research is warranted to evaluate the clinical potential of these drugs and compounds.

Despite the significant findings of our study, it is important to acknowledge the limitations inherent in our research. Firstly, it is crucial to recognize that our study primarily relied on bioinformatics analysis, and further validation through clinical or experimental studies is necessary to confirm the robustness and reliability of our results. It is important to conduct basic experiments to gain a deeper understanding of the functional roles of the identified hub genes and their underlying regulatory mechanisms in the context of osteoporosis. Secondly, it is worth noting that the sample tissues utilized in our study were derived from circulating monocytes rather than bone tissues. Although monocytes have been shown to exhibit potential as a surrogate for bone cells in certain studies, it is essential to obtain more detailed clinical characteristics and conduct further investigations using bone tissue samples to validate the performance of our diagnostic model accurately. Thirdly, it is important to acknowledge that the characteristics and risk factors associated with osteoporosis vary across different ethnic groups and environmental contexts. Therefore, it is crucial to evaluate the validity and effectiveness of machine learning models in diverse populations worldwide.

## Conclusion

Our study focused on characterizing the transcriptomic landscape of the Wnt signaling pathway in postmenopausal Caucasian women with osteoporosis. By leveraging machine learning analysis of Wnt pathway-related genes, we successfully developed and validated a highly accurate diagnostic model, exceeding the performance of existing methods. This novel approach paves the way for personalized screening and earlier intervention in osteoporosis, potentially reducing fracture risk and improving patient outcomes. Our identification of hub genes within the Wnt pathway sheds light on previously unknown regulatory mechanisms in osteoporosis. Targeting these genes, alone or in combination, holds promise for the development of novel therapeutic strategies. In conclusion, our study delivers significant advancements in osteoporosis research by integrating bioinformatics and machine learning methodologies. The high-precision diagnostic model and identified therapeutic targets offer exciting possibilities for early diagnosis, personalized treatment, and ultimately, improved quality of life for osteoporosis patients.

### Supplementary Information

Supplementary Information.

## Data Availability

The datasets involved in this study are available in the GEO database (https://www.ncbi.nlm.nih.gov/geo/), accession numbers: GSE56814, GSE56815, GSE2208, GSE7158, GSE7429, and GSE13850.
