# Supplementary material for: Investigating the impact of Wnt pathway-related genes on biomarker and diagnostic model development for osteoporosis in postmenopausal females
Source: Sci Rep. 2024 Feb 4;14:2880. doi: 10.1038/s41598-024-52429-1 (PMC10838932; doi:10.1038/s41598-024-52429-1)
Supplement: Supplementary file 1 — Supplementary Information. [file 41598_2024_52429_MOESM1_ESM.docx]

**Figure S1**


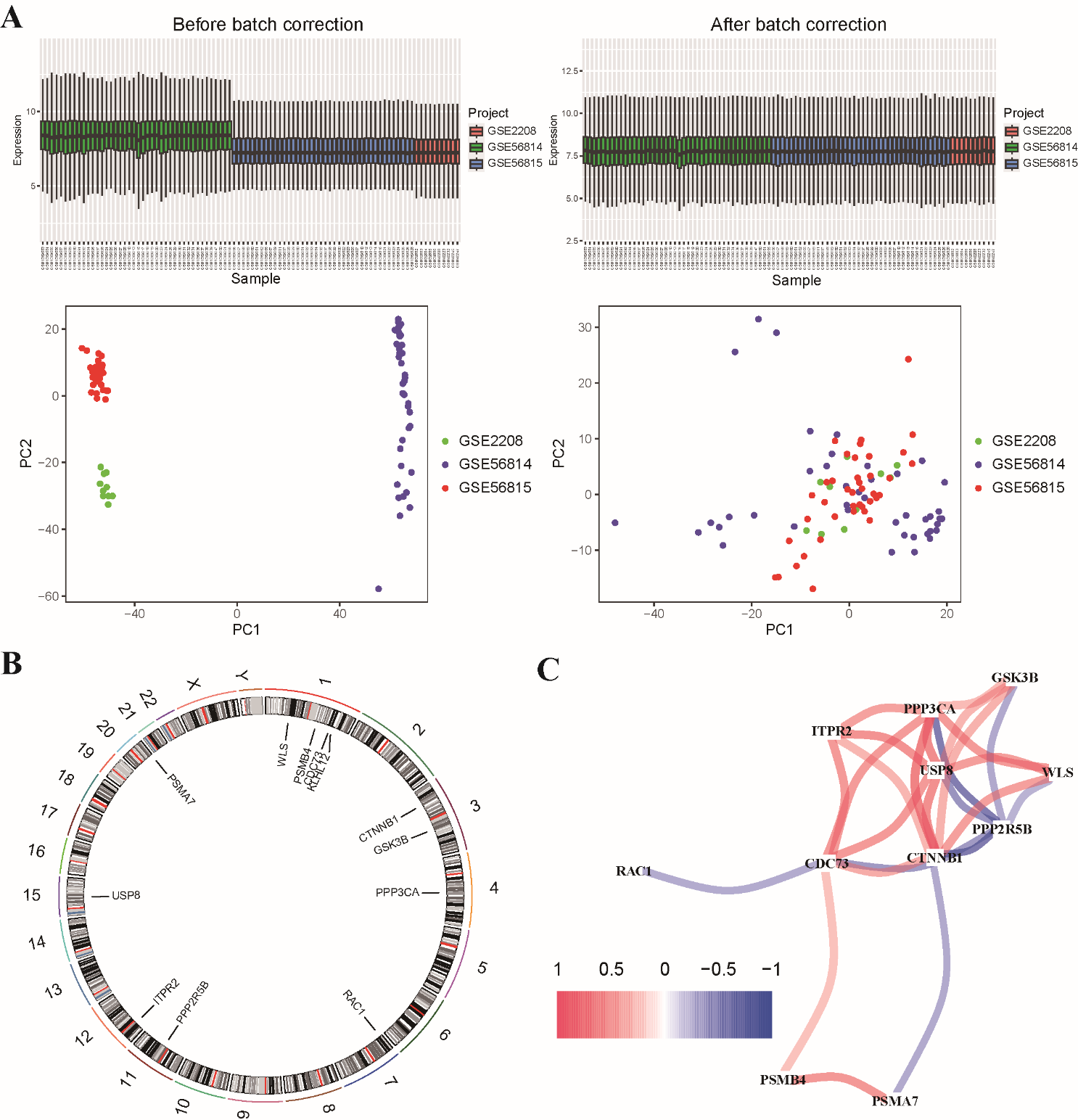


**Figure S1. (A)** Principal Component Analysis (PCA) was employed to reduce dimensionality in expression profiles across all samples, both before and after dataset processing. (**B)** The chromosomal positions of the 12 osteoporosis-associated Wnt pathway-related genes were shown in the graph. (**C)** The network diagram depicting the relationships among these osteoporosis-associated Wnt pathway-related genes, KLHL12 does not exhibit correlations with any other gene (Absolute correlation coefficient cutoff = 0.3).

**Figure S2**

**
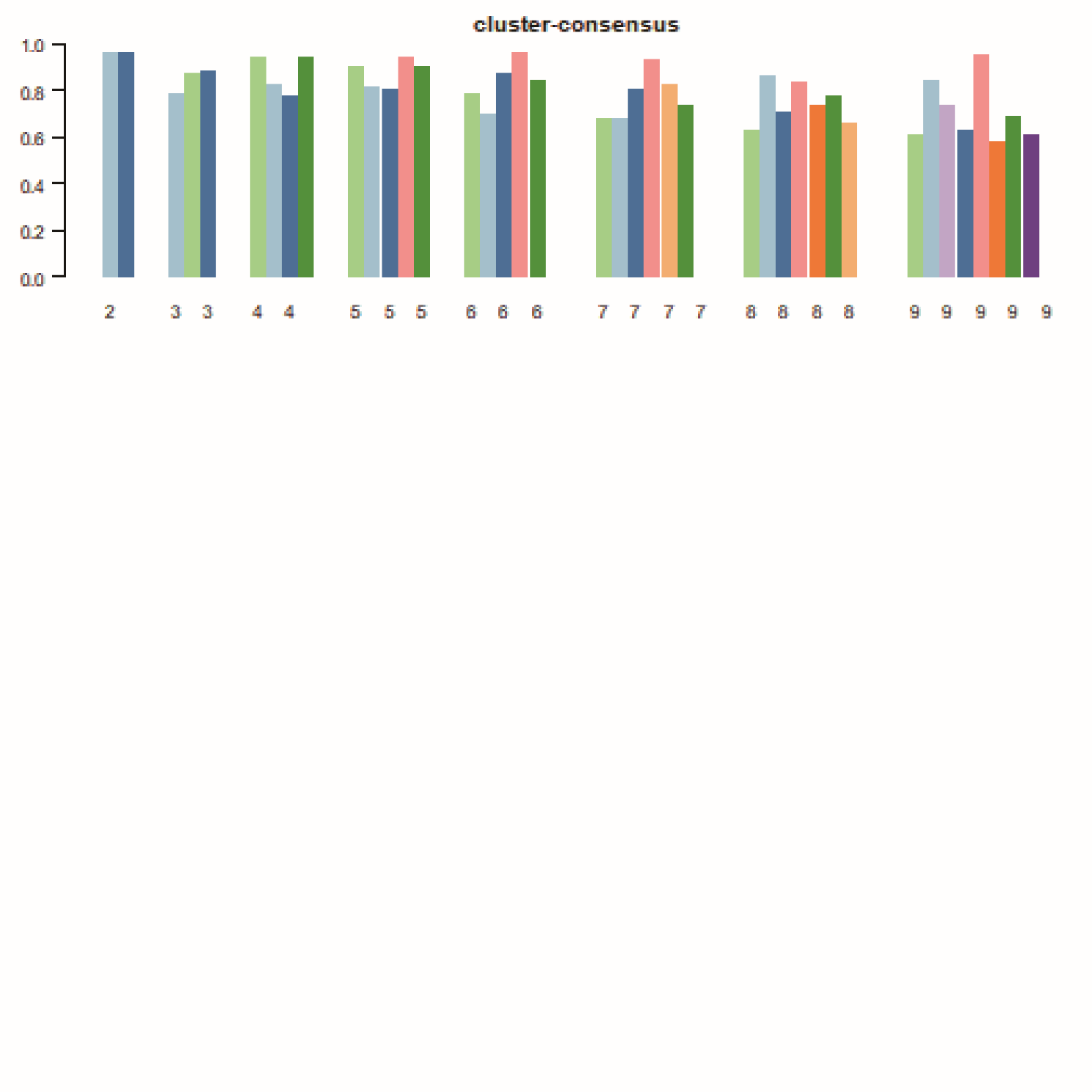
**

**Figure S2.** The radial chromosomal positions of these 11 genes were illustrated in a loop graph.

Table S1 Basic characteristics of training datasets for monocyte microarray analyses.

|  |  | High BMD | |  |  | Low BMD |  |
| --- | --- | --- | --- | --- | --- | --- | --- |
| Datasets | N | Age | Hip BMD Z score |  | N | Age | Hip BMD Z score |
| GSE56814 | 26 | 54.0 (1.8) | 1.28 (0.46) |  | 16 | 52.6 (2.5) | -1.17 (0.60) |
| GSE56815 | 20 | 57.2 (1.9) | 1.58 (0.66) |  | 20 | 20 | -1.04 (0.38) |
| GSE2208 | 5 | 52.6 (1.8) | 2.22 (0.93) |  | 5 | 51.4 (1.5) | -1.69 (0.16) |
